# The use of poly(styrene-*block*-isobutylene-*block*-styrene) as a microshunt to treat glaucoma

**DOI:** 10.1093/rb/rbw005

**Published:** 2016-02-25

**Authors:** Leonard Pinchuk, Isabelle Riss, Juan F. Batlle, Yasushi P. Kato, John B. Martin, Esdras Arrieta, Paul Palmberg, Richard K. Parrish, Bruce A. Weber, Yongmoon Kwon, Jean-Marie Parel

**Affiliations:** ^1^InnFocus, Inc., Miami, FL, USA;; ^2^Ophthalmic Biophysics Center, Bascom Palmer Eye Institute, University of Miami Miller School of Medicine, Miami, FL, USA;; ^3^Pôle Ophtalmologique De La Clinique Mutualiste, Pessac Cedex, France;; ^4^Centro Laser, Santo Domingo, Dominican Republic;; ^5^Anne Bates Leach Eye Hospital, University of Miami, Bascom Palmer Eye Institute, Miller School of Medicine, Miami, FL, USA and; ^6^Department of Ophthalmology, University of Paris Hotel-Dieu Hospital, Paris, France

**Keywords:** biocompatibility, medical device, stent, soft tissue

## Abstract

The InnFocus MicroShunt® is a minimally invasive glaucoma drainage microtube used to shunt aqueous humor from the anterior chamber of the eye to a flap formed under the conjunctiva and Tenon’s capsule. The safety and clinical performance of this device approaches that of trabeculectomy with mitomycin C, the current ‘gold standard’ treatment for advanced glaucoma. The invention of a new biomaterial called poly(styrene-*block*-isobutylene-*block*-styrene) or ‘SIBS’ is the enabling factor which led to the success of this product. SIBS is ultrastable with virtually no foreign body reaction in the body, which manifests as clinically insignificant inflammation and capsule formation in the eye. The lack of capsule formation enables unobstructed flow through the 70 µm lumen tube and the achievement of controlled low intraocular pressure, which is important for the management of glaucoma. This article summarizes the integration of SIBS into a glaucoma drainage device and confirms its functionality with clinical success over a 2-year period.

## Introduction

### The development of poly(styrene-*block*-isobutylene-*block*-styrene)

Work on a new biomaterial began in the early 1990s when Dr Leonard Pinchuk observed that conventional polyether urethane implantable biomaterials, such as those comprising the insulators on pacemaker leads and the synthetic vascular graft being developed by his team at Corvita Corporation (Miami, FL), persistently attracted granulocytes as a consequence of their slow unintended biodegradation in the body. Macrophages, polymorphonuclear leukocytes and foreign body giant cells migrated toward the device to either wall-off the degrading material by forming thick capsules around it or to disperse degraded fragments by phagocytosis. Many pacer lead insulators were and still are made from industrial-grade polyether urethanes that were never designed for long-term applications in the body [1]. In an effort to produce a synthetic vascular graft, Dr Pinchuk’s team set out to develop a biostable polymer with no sites for degradation—i.e., no urethane, ether, ester, carbonate, carbamate, amide and so on—linkages, on either the backbone or side groups of the polymer. Fortunately, a family of such materials had been developed a decade earlier by Kennedy and Ivan [2] at the University of Akron (Akron, Ohio). The University of Akron scientists were unaware of the biodegradation issues that plagued the implant industry and the synthesis of the elastomer was never stepped-up or purified for implant applications. Corvita immediately licensed this family of polymers for medical applications and applied for additional patents to protect new discoveries and applications [3, 4]. The Corvita team then developed equipment and processes for stepping-up and purifying the reactions for implantable applications.

**Figure 1.**
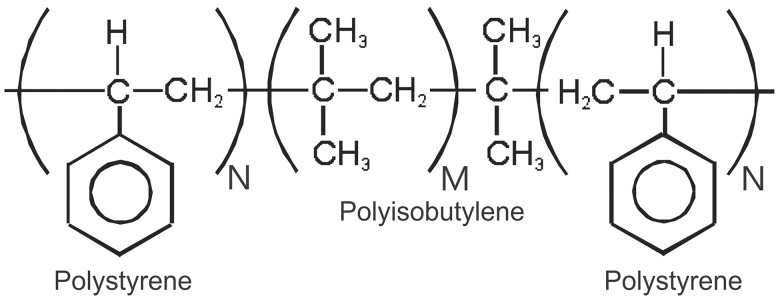
A simplified chemical structure of SIBS with a central block that comprised polyisobutylene (incorporated initiator not shown) and end blocks of polystyrene (*M* is an integer >*N*)

The key feature in the Akron materials was the base copolymer, in the simplified central block of the triblock polymer in Fig. 1. Polyisobutylene itself is a gum resembling chewing gum. Polyisobutylene does not contain any of the aforementioned labile linkages, and better still, there is a dimethyl group on every second carbon which prevents the backbone from oxidizing to form double bonds—the bane of many polyolefins such as polyethylene and polypropylene. The presence of double bonds on the backbone of polymers leads to embrittlement, low flex fatigue life and degradation. The conversion of polyisobutylene into an elastomer requires crosslinking either with permanent crosslinks or meltable pseudocrosslinks. In order to process the material into moldable or extrudable medical devices, meltable glassy blocks comprised of polystyrene were polymerized onto both ends of the polyisobutylene central block to bind the amorphous (elastic/rubbery) polyisobutylene segments together. The triblock polymer, poly(styrene-*block*-isobutylene-*block*-styrene) or ‘SIBS’, is shown in Fig. 1, where *N* is an integer greater than *M*.

The first medical use of SIBS was for Boston Scientific Corporation’s (Natick, MA) TAXUS® stent [5, 6]. TAXUS is a small balloon-expandable metallic stent (2–3 mm in diameter and 10–20 mm long), with a permanent SIBS coating that slowly releases the antiproliferative drug paclitaxel into the wall of the coronary artery to prevent restenosis. TAXUS became the largest product launch in the history of medical devices with sales of ∼$3 billion in the first year. Data collected from studies of TAXUS confirmed no biodegradation and minimal tissue reaction [7]. This interaction of Dr Pinchuk’s team with Boston Scientific led Boston Scientific to provide the seed money to establish InnFocus LLC which was formed in 2004. InnFocus LLC was converted to InnFocus, Inc. in 2011. The goal of InnFocus is to develop products made from SIBS for use in the eye. A glaucoma shunt was the first product.

### Development of the glaucoma shunt

Sometime around 2003, Dr Pinchuk introduced SIBS to Dr Jean-Marie Parel at the University of Miami’s Miller School of Medicine, Bascom Palmer Eye Institute, Optical Biophysics Center (OBC). Dr Parel’s collaborators implanted 3-mm diameter, 1-mm thick SIBS disks in the corneal stroma as well as under the conjunctiva and Tenon’s capsule in the eyes of New Zealand White Rabbits. Similar disks made from silicone rubber (polydimethylsiloxane) were implanted alongside the SIBS disks as controls. The results of the 2-month implants were published by Parel *et al*. [8] and Acosta *et al*. [9] and, in brief, they found that there were no myofibroblasts or angiogenesis in the vicinity of the SIBS disks, nor were there integral capsules surrounding the disks. In contrast, the silicone rubber controls showed angiogenesis, myofibroblasts and significant capsules attached to the disks. In summary, SIBS was found to be totally innocuous in the eye.

Shortly thereafter, Dr Pinchuk and Dr Parel met with Dr Francisco Fantes, a glaucoma surgeon, to determine how best to exploit this nonencapsulating SIBS material. It was decided that a glaucoma drainage device without a plate might be achievable if the tube did not occlude. Preventing the tube from clogging requires that the lumen be larger than the diameter of a sloughed endothelial cell, which is about 40–50 µm, while at the same time sufficiently small to prevent hypotony (low intraocular pressure (IOP) that can traumatize the eye). The lumen size was approximated from the Hagan–Poiseuille equation and a series of rabbit eye implants by Arrieta *et al.* [10, 11] confirmed that a lumen diameter of ∼70 µm would satisfy these requirements. It was also decided that draining to a flap under the conjunctiva and Tenon’s capsule (to a bleb), similar to the ‘gold standard’ trabeculectomy, made the most sense. This rationale for draining to a bleb is explained in more detail by Pinchuk *et al*. [12]. The advantage of the MicroShunt would be the avoidance of cutting the sclera and suturing the scleral flap with sutures placed under the proper tension to control outflow, a process that requires significant surgical skill. In addition, the fluid dynamics of the MicroShunt could be controlled by the lumen diameter and length to minimize hypotony. And so began the development of a SIBS-based microshunt [13].

There were three major iterations of shunt design that were tested first in chronic rabbit eye studies at the University of Miami, Bascom Palmer Eye Institute OBC laboratory, and then in pilot feasibility studies over a period of 4 years to determine the best design as well as the best implant technique [12]. All animal studies were authorized by the University of Miami Animal Care and Use Committee.

**Figure 2.**
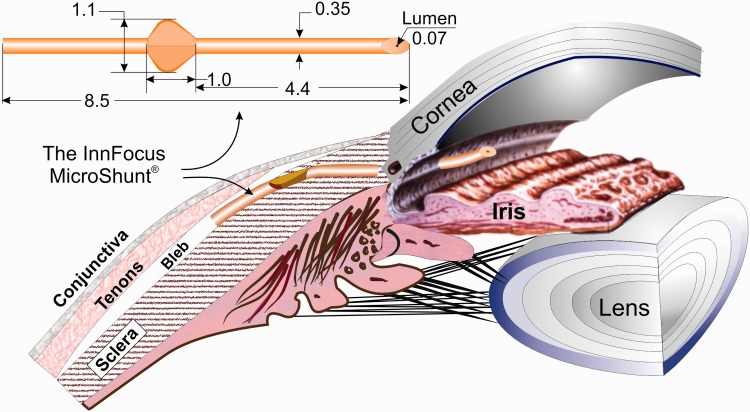
The InnFocus MicroShunt showing its dimensions (mm) and placement under the limbus with its proximal end in the anterior chamber and distal end under the conjunctiva and Tenon’s capsule

The final design, currently used in clinical studies, called the InnFocus MicroShunt®, is shown in Fig. 2 along with its location in the eye The MicroShunt consists of an 8.5-mm long SIBS tube with an outer diameter of 350 µm and a lumen diameter of 70 µm. Located half-way down the tube is a planar fixation member resembling the fins on an arrow which serves (i) as a ‘cork’ to seal the device in the pocket and prevent leakage around the tube; (ii) as a stopper to prevent the device from migrating into the eye and (iii) as a mechanism of orienting the device such that the bevel in the anterior chamber faces the cornea such that the entrance to the lumen can be cleared if blocked by debris.

## Methods

After approval of the implant protocol by CONABIOS (the Dominican Republic National Counsel of Bioethics and Health) and the local hospital-based ethics committee, a prospective study was conducted by Dr Juan F. Batlle and his team at Centro Laser, Santo Domingo, Dominican Republic. The major inclusion criteria included primary open angle glaucoma (POAG) patients who failed maximum tolerated glaucoma medication. Combined patients undergoing cataract surgery were allowed in this study but were not a requirement. Patients who had failed previous conjunctiva surgeries were excluded from the study. All eligible patients in the practice of the principal investigator, who would otherwise be considered for primary trabeculectomy, were offered participation in the study; none refused and all signed the appropriate consent forms. The patients were enrolled prospectively in the order of consent.

**Figure 3.**
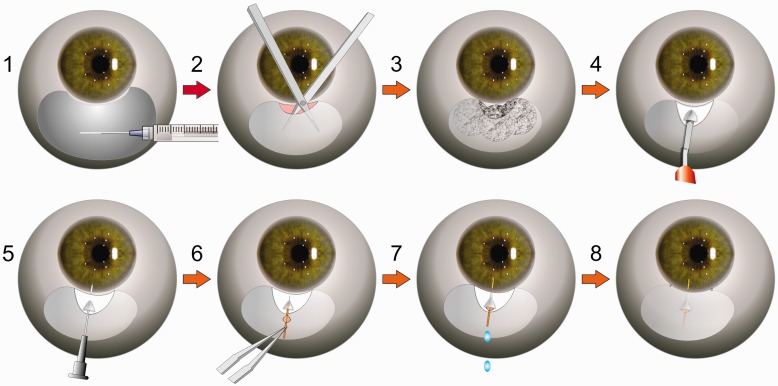
Schematic of implantation procedure: (i) an anesthetic is injected under the conjunctiva; (ii) an incision is made below the limbus and a flap dissected above the sclera with a blunt scissor; (iii) three sponges saturated with mitomycin C are placed in the flap for ∼3 min followed by irrigation with sterile saline solution; (iv) a shallow 1 mm wide, 1–2 mm deep scleral pocket is formed 3 mm below the limbus; (v) a 25-G needle is advanced through the scleral pocket into the anterior chamber; (vi) the MicroShunt is maneuvered through the scleral pocket and needle tract with a forceps and the fins of the device wedged snugly into the scleral pocket; (vii) flow of aqueous humor from the anterior chamber to the flap is confirmed by drop observation and (viii) the distal end of the device is tucked under the conjunctival/Tenon’s flap and the flap closed with multiple interrupted 10-0 nylon sutures

The MicroShunt was provided by the manufacturer InnFocus, Inc. (Miami, FL) in a sterile packaged kit which contained (i) a ruler to measure the site of entry (3 mm from the limbus); (ii) a marking pen to ink the ruler; (iii) three LASIK sponges to apply mitomycin C (MMC) (0.4 mg/ml for 3 min (MMC not supplied)); (iv) a 1 mm × 1 mm triangular knife to incise a shallow pocket in the sclera and (v) a 27 or 25G needle to form a needle tract under the limbus to the anterior chamber. The implant procedure is shown in Fig. 3. A light pressure patch was used the day after surgery and nightly for 5 days thereafter.

## Results

Baseline demographic characteristics are described in Table 1. Twenty-one phakic (still had their natural lens) patients (11 with visually impairing cataracts) and 2 pseudophakic (had intraocular lens implants) patients participated in the study. The mean baseline IOP on full medication was 23.8 ± 5.3 mm Hg (range 19–38 mm Hg) in the study eye. All 23 patients had POAG with IOP that met the inclusion criteria. The mean baseline best corrected distance visual acuity (VA) was 20/60 (range 20/20 to light perception).

**Table 1.** Summary of baseline characteristics of patients prior to implantation of the InnFocus MicroShunt

Number of patients	23
Average age	59.8 ± 15.3
Race	Mixture of African, native aborigine and white
Status of test eye: phakic/cataract/pseudophakic	10/11/2
Glaucoma diagnosis	23 POAG
Previous conjunctival surgeries	None
Baseline IOP (with full medication regimen)	23.8 ± 5.3 mm Hg
Average glaucoma medications/patient	2.4 ± 1.0
Visual field mean deviation average	−20.1 ± 12.1 dB

**Figure 4.**
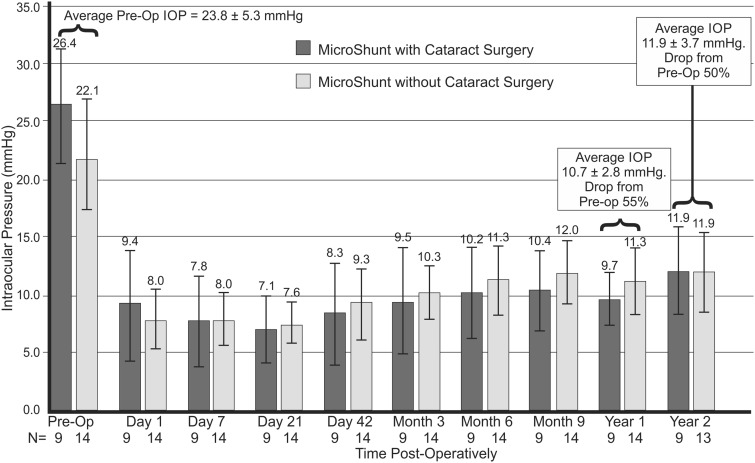
Change in intraocular pressure (mm Hg) with time for the MicroShunt implanted with and without cataract surgery

**Figure 5.**
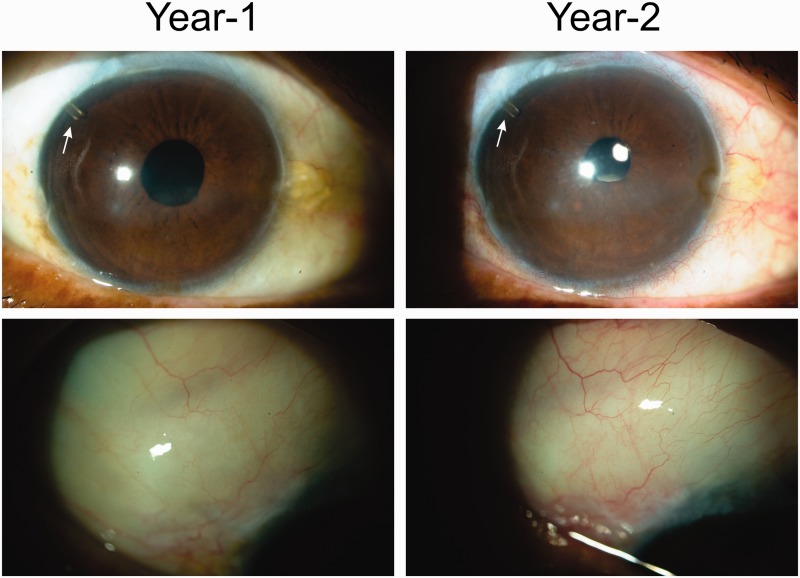
Typical eye with the InnFocus MicroShunt at 1 and 2 years. The white arrows in the upper photographs point to the proximal end of the MicroShunt in the anterior chamber

Postoperative data were available for 23 patients at 1 year and 22 patients at 2 years, as 1 patient was lost to follow-up. Fourteen patients underwent MicroShunt implantation alone and 9 underwent combined phacoemulsification with subsequent IOL implantation followed by MicroShunt insertion. A summary of the 1- and 2-year data is presented in Table 2. A bar chart of IOP with time is presented in Fig. 4. The qualified success rate as defined by IOP ≤ 18 mm Hg and with ≥20% reduction in pressure from baseline was 100% at 1 (23/23) and 2 years (22/22). Figure 5 shows images of the eye with the MicroShunt at 1 and 2 years showing the entrance of the SIBS tube (see arrows) and the nature of the blebs.

**Table 2.** Summary of 1- and 2-year results for the InnFocus MicroShunt

Follow-up time	Year 1	Year 2
Number of patients	23	22
Intraocular pressure (mm Hg)	10.7 ± 2.8	11.9 ± 3.7
Percent IOP reduction from baseline	55	50
Average glaucoma medications/patient	0.3 ± 0.8	0.4 ± 1.0
Percent of patients totally off of eye drops	87%	86%
Qualified success as defined by IOP ≤ 18 mm Hg and a drop in IOP ≥ 20%	100%	100%

There were no VA losses or gains >1 line in any of the patients who had glaucoma surgery alone over the 2-year time frame tested. There were 3 patients who gained 2 or more lines at 1 year, and 4 patients at 2 years, following implantation of a MicroShunt in combination with cataract surgery.

The most common postoperative adverse events were mild and consisted of IOP < 5 mm Hg after day 1, which occurred in three combined cases (3/23 (13.0%) of all cases) and all resolved spontaneously by day 90. Shallow anterior chambers were observed in 3 of the 23 (13.0%) patients; however, no patient required reformation of the anterior chamber or drainage of a choroidal effusion. Choroidal detachment was observed in 2 patients (8.7%) from the combined group which resolved spontaneously. There were no sight-threatening long-term adverse events.

## Discussion and conclusions

The development of SIBS and subsequently the InnFocus MicroShunt was an educated iterative process that occurred over the course of 20 years [5, 12]. The process required sophisticated chemistry and engineering including controlling the foreign body reaction with SIBS, designing the shunt to be atraumatic with a lumen size that minimized hypotony and developing a design and implant procedure that protected the conjunctiva from being eroded by the device. Fins on the shunt are held firmly in the shallow pocket formed in the sclera and act as a cork to divert aqueous humor into the lumen of the device, which due to its hydrodynamic design, minimized hypotony. Draining to the conjunctival/Tenon’s flap (also known as a bleb), as does the gold standard trabeculectomy, is important as the shunt bypasses the high resistances that can be anywhere in the drainage path for aqueous humor, e.g., the trabecular meshwork, Schlemm’s Canal, the collector channels, the aqueous veins and the episcleral veins. Drainage of aqueous humor from the bleb follows the path of least resistance, which could include drainage into the episcleral venous system or by percolating through the microcysts in the conjunctiva and into the tear film, or both. Microcysts are naturally occurring channels that form in the conjunctiva [14].

The InnFocus MicroShunt is effective in lowering IOP by 50–55% and in significantly reducing the need for glaucoma medications, with no long-term adverse events. The control of IOP to a level below 14 mm Hg in over 80% of patients suggests that glaucomatous progression of vision loss will be unlikely [15]. The advantages of the MicroShunt procedure include the following: (i) no dissection of the sclera; (ii) ease of procedure without the need for special equipment; (iii) no reliance on subjective suture tension; (iv) the ability to place several devices in the same eye and (v) minimal need for postoperative interventions (such as suture lysis).

The enabling factor that led to the success of the MicroShunt is the use of SIBS as the tube comprising the shunt. SIBS itself provokes clinically insignificant inflammation and encapsulation. In addition, it is soft with a low modulus and due to its thermoplastic nature conforms to the curvature of the eye and eventually creeps into a stable nonirritating and noneroding configuration. The thermoplastic nature of SIBS can be contrasted to the thermoset nature of silicone rubber tubes which tend to straighten in the eye which can erode the conjunctiva—this is the reason why silicone rubber tubes used in large drainage devices in the eye often require a patch graft over the tube to prevent erosion [16]. SIBS is also tough which enables the bleb to be repaired; that is, if the bleb fails by healing closed, it can be opened by ‘needling’ without damaging the device [17]. Similarly, if the entrance to the device becomes occluded with debris, the debris can be vaporized with a laser without damaging the device. Finally, the InnFocus MicroShunt is sufficiently small that many devices can be placed in the same eye in the event one fails or if a lower target pressure is desired.

The intended use of the InnFocus MicroShunt is to provide a simple alternative to primary trabeculectomy. Once its safety and effectiveness are well established, it is expected that this device will be used in the treatment of earlier stage patients as an alternative to long-term glaucoma medication where the drugs, or rather the preservatives in the drugs [18], can wreak havoc on the cornea as well as the conjunctiva and severely limit treatment effectiveness in the future.

The InnFocus MicroShunt was CE Marked on 9 January 2012 in Europe and several other clinical studies are under way in Europe, Asia and Canada to increase the number of patients and to investigate limitations of the device. In addition, a US Investigational Device Exception was granted by the Food and Drug Administration (FDA) in May 2013 and a multicenter clinical trial comparing the MicroShunt to primary trabeculectomy in patients refractory to medication is under way.
